# Perforation of Right Coronary Artery During Coronary Angioplasty: A Rare Complication

**DOI:** 10.7759/cureus.25278

**Published:** 2022-05-24

**Authors:** Rahul Navab, Sambasiva Reddy N

**Affiliations:** 1 Internal Medicine, People’s Education Society Institute of Medical Sciences and Research, Kuppam, IND; 2 Interventional Cardiology, People's Education Society Institute of Medical Sciences and Research, Kuppam, IND

**Keywords:** echocardiogram, cardiac chamber, angioplasty, right coronary artery (rca), perforation

## Abstract

Coronary angioplasty procedure, also known as percutaneous transluminal coronary angioplasty (PTCA), is performed to restore blood flow across significantly blocked coronary vessels. Perforation of coronary vessels may occur rarely during the procedure or within 24 hours post-procedure and is considered a serious complication. We wish to share our experience of a case of perforation in the proximal and mid-portion of the right coronary artery (RCA) during coronary angioplasty. To seal the perforation, the balloon was inflated and vitals were monitored. Check coronary angiography showed persisting extravasation but with no collection on serial echocardiograms. It was confirmed that the perforation was not in the pericardial space but inside the cardiac chamber. The patient was shifted to the cardiac care unit, for further monitoring of vitals and echocardiogram studies for the next 72 hours to ensure recovery. Wire-induced coronary perforations into the cardiac chamber are most of the time benign and are conservatively managed.

## Introduction

Coronary angiography is a minimally invasive procedure that allows interventional cardiologists to detect blocked or narrowed vessels in the heart. Perforation of the coronary artery during coronary angioplasty is a serious complication but is seldom encountered in the cardiac catheterization laboratory. The incidence of coronary artery perforation ranges from 0.1%-0.71% with mortality varying from 7% to 17% [1]. Most often, the devices that lead to perforation are intracoronary balloon, guidewire, rotablation, and directional atherectomy [2]. Several treatment options are available for this complication, including prolonged balloon dilatation, use of coronary stent graft, and bypass surgery [3].

## Case presentation

**Figure 1:**
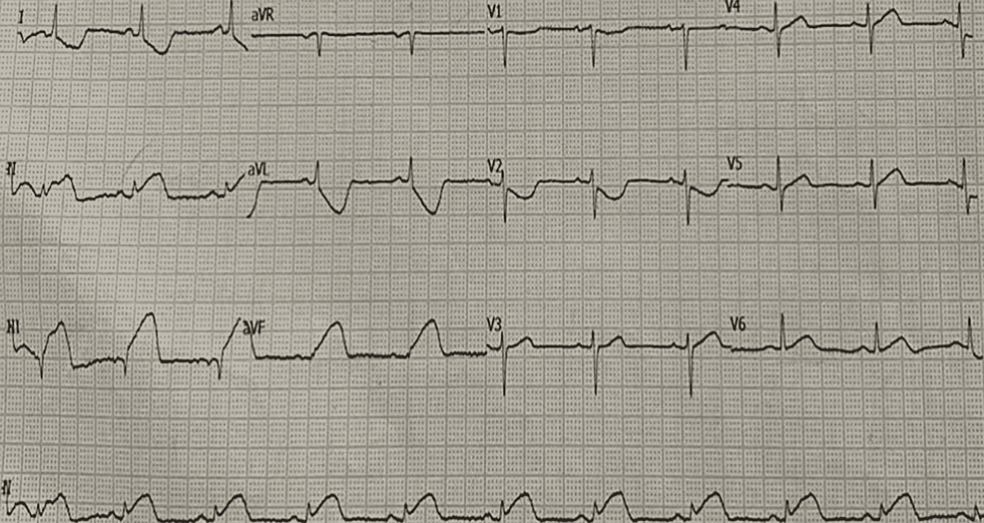
ECG frame showing ST-segment elevation

**Figure 2:**
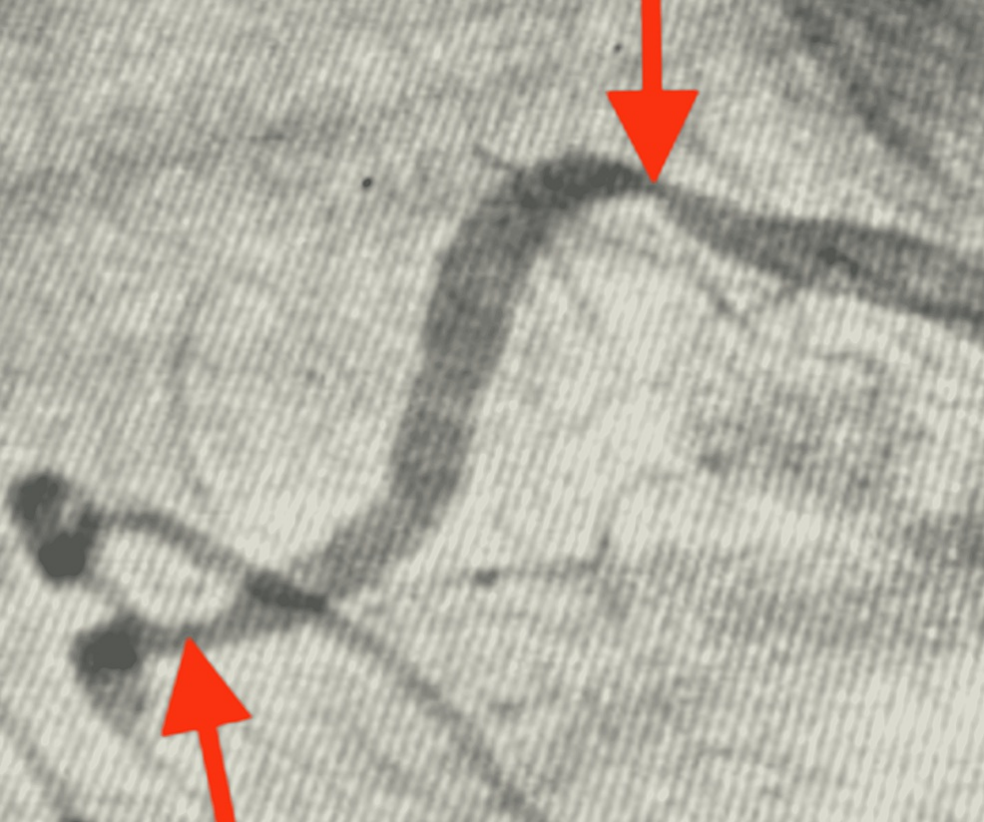
Angiographic frame revealing stenosis at proximal and mid-portion of RCA (red arrows) RCA - right coronary artery

**Figure 3:**
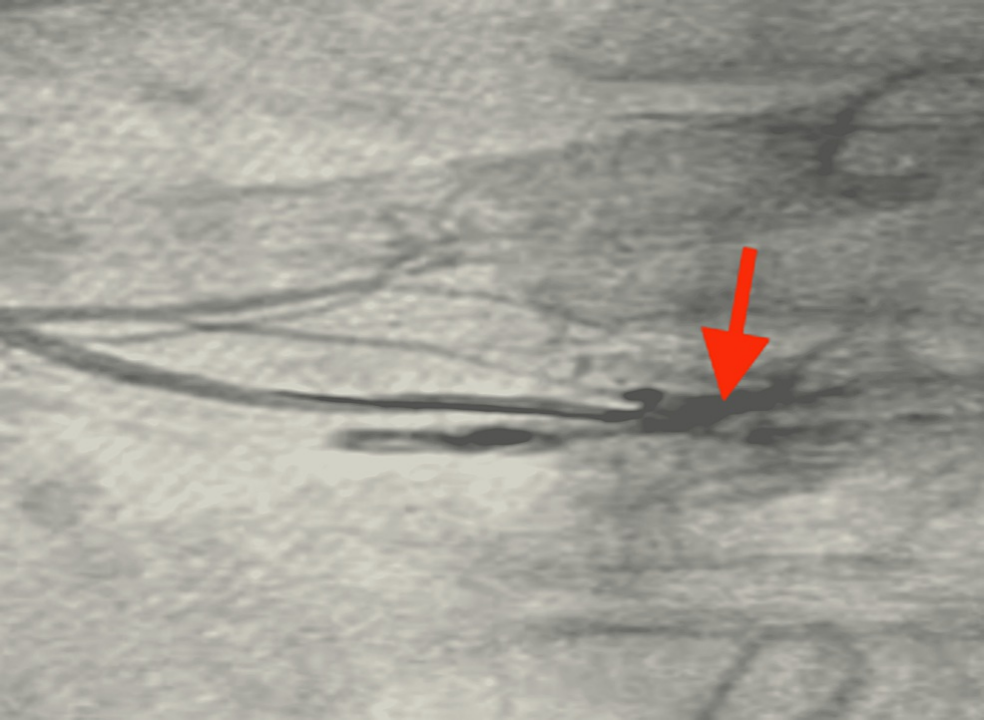
Angiographic frame showing perforation (red arrow)

**Figure 4:**
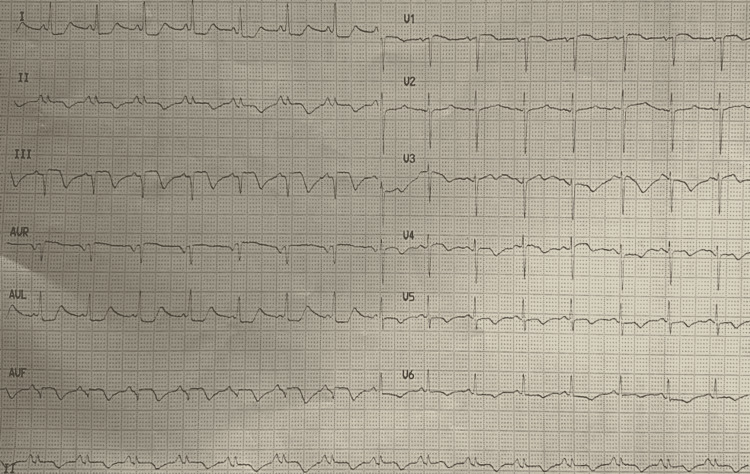
Post PTCA procedure ECG frame showing ST-segment depression and T-wave inversion PTCA - percutaneous transluminal coronary angioplasty

A 65-year-old-female with a history of hypertension for 20 years was admitted with a complaint of chest pain, retrosternal, radiating to the left side of the neck and inter-scapular area. Heart rate (HR) was 82 beats per minute (bpm) and regular. Her blood pressure was 128/86 mmHg. The baseline 12-lead ECG revealed infero-posterior wall ST-elevation myocardial infarction (STEMI) (Figure 1). Echocardiography revealed normal chamber dimensions with base and mid inferior, infero-septal wall hypokinesia with an ejection fraction of 50%, mild left ventricular systolic dysfunction, and grade-1 left ventricle diastolic dysfunction. Subsequently, coronary angiogram was planned and it revealed 70% - 99% stenosis in the proximal and mid-portion of the right coronary artery (RCA) respectively (Figure 2). As it was an inferior wall STEMI, the main culprit was the RCA. The right femoral artery was accessed, a fielder fine control guidewire was gently pushed into the artery through the needle and a 7F (French) catheter was passed through it to the ostium of RCA. Guidewire was placed in distal RCA. A non-ionic contrast dye was injected into the coronary arteries. A non-complaint balloon of 3.0 x 12 mm length was advanced over the guidewire and the balloon was inflated up to 18 atm (standard atmosphere). After pre-dilatation, two Xience prime stents (Abbott Vascular, Santa Clara, California) ranging from 3.0 x 23 mm - 3.5 x 28 mm diameter were placed and deployed over the proximal and mid-portion of RCA at a pressure of 12 atm - 14 atm. Extravasation of contrast dye was noted and therefore, perforation was confirmed (Figure 3). To prevent leaks into the pericardium and also to seal the perforation, balloon dilatation was done. Vitals were continuously monitored and the patient was found to be hemodynamically stable. Check angiography revealed persisting leak from the perforation site and no collection was noted in the pericardial space. Serial echocardiograms were done and it showed no signs of pericardial fluid. It was confirmed that the perforation was not in the pericardial space but inside the cardiac chamber. Post-percutaneous transluminal coronary angioplasty (PTCA) procedure ECG revealed ST-segment depression in lead I, AVL, and T-wave inversion in lead II, lead III, AVF, V4, V5, and V6 (Figure 4). The patient was shifted to the cardiac care unit; vitals were continuously monitored for the next 72 hours and the patient was discharged.

## Discussion

A coronary angiogram is a gold standard diagnostic test done to visualize the blood flow in the coronary arteries around the heart and angioplasty is a procedure that is done to unblock coronary arteries which are narrowed due to plaque or blood clots. Stenting improves blood flow and also relieves heart disease symptoms.

A coronary artery perforation can be caused by various coronary intervention procedures, including injury from guidewires, with oversized balloons or athero-ablative devices used in angioplasty, as well as stent placement [4].

Ellis et al. classified coronary artery perforations into three types according to coronary angiographic findings [5] (Table 1).

**Table 1:** Classification of coronary artery perforations (CAP) based on coronary angiographic findings by Ellis et al. [5]

Classification of coronary artery perforations	
Type 1	Extraluminal crater without extravasation
Type 2	Pericardial or myocardial blush without contrast jet extravasation
Type 3	Extravasation through frank (> or =1 mm) perforation and cavity spilling

According to the above-mentioned classification, this case comes under type 3 coronary artery perforation. Older age, previous history of heart disease, diabetes, and female gender are the risk factors for coronary artery disease. A perforation caused by guide wires is often silent at first but can progress to tamponade as a result of hypotension or chest pain [6]. The clinical outcome can range from spontaneous closure of the perforation to cardiac tamponade, which can be fatal [4]. In this case following stent placement, check angiography revealed extravasation of dye during the procedure, confirming perforation of the coronary artery.

One or more of the following criteria defines pericardial tamponade [7] (Table 2).

**Table 2:** Criteria for defining pericardial tamponade

Criteria for pericardial tamponade	
A	systemic hypotension (systolic blood pressure less than 90 mm/hg)
B	fluid collection in the pericardium, major respiratory change in trans-mitral velocity of doppler, inspiration leads to the collapse of dilated inferior venacava detected on echocardiogram
C	pericardial space is filled with a significant collection of dye detected by angiogram

All the above-mentioned findings were not found in this case; the patient's vital signs were stable and there was no shortness of breath or tachycardia. The hemodynamic compromise did not occur in this case. To seal the perforation, prolonged balloon inflation was done. Serial echocardiograms were done, which revealed no pericardial effusion. But the extravasation was persisting and it was confirmed that perforation was into the cardiac chamber. She was under conservative management and shifted to the cardiac care unit for further monitoring. Usually, perforations of the coronary artery into pericardial space are managed by occluding the vessel with a balloon, using a covered stent, distal fat, or coil embolization, and on rare occasions by emergency surgery [8].

## Conclusions

Coronary artery perforation during an angioplasty procedure is a rare but dangerous complication that can lead to an increase in morbidity and mortality. Early recognition and appropriate management are crucial in reducing complications during the procedure. Guidewire-induced perforation into the cardiac chamber does not require any intervention most of the time. However, the availability of new equipment devices and the experience of interventionalists with the devices can decrease the complications.
